# Supplementary material for: Scalable and Consolidated Microbial Platform for Rare Earth Element Leaching and Recovery from Waste Sources
Source: Environ Sci Technol. 2023 Dec 27;58(1):570–9. doi: 10.1021/acs.est.3c06775 (PMC10785750; doi:10.1021/acs.est.3c06775)
Supplement: Supplementary file 1 — es3c06775_si_001.pdf [file es3c06775_si_001.pdf]

Supporting Information

A scalable and consolidated microbial platform for rare earth element leaching and recovery from waste sources

Nathan M. Good[†], *Christina S. Kang-Yun*[‡], *Morgan Z. Su*[†], *Alexa M. Zytnick*[†], *Colin C. Barber*[†], *Huong N. Vu*[∦], *Joseph M. Grace*[∦], *Hoang H. Nguyen*[∦], *Wenjun Zhang*[#], *Elizabeth Skovran*[∦], *Maohong Fan*[&], *Dan M. Park*[‡], *Norma Cecilia Martinez-Gomez*[†,*]

† Department of Plant and Microbial Biology, University of California, Berkeley, Berkeley, California 94720, USA

Department of Chemical and Biomolecular Engineering, University of California, Berkeley, Berkeley, California 94720, USA

‡ Physical and Life Sciences Directorate, Lawrence Livermore National Laboratory, Livermore, California 94550, USA

∦ Department of Biological Sciences, San José State University, San José, California 95192, USA

& Department of Chemical and Biomedical Engineering, University of Wyoming, Wyoming 82071, USA

*Address correspondence to N. Cecilia Martinez-Gomez, cecimartinez@berkeley.edu

Number of Pages: 18

Number of Figures: 9

Number of Tables: 2

SUPPLEMENTAL METHODS

Hypho medium optimization. *M. extorquens* AM1 pre-cultures were grown as described. Cells were pelleted by centrifugation at 1,000 x *g* for 10 minutes, washed in 1 mL sterile Hypho medium, and then resuspended in 200 µL sterile Hypho medium. 48-well tissue culture plates (Corning, New York) were prepared with 640 µL of medium prepared with standard phosphate (P_i; 2.53 g/L K_2HPO_4, 2.59 g/L $NaH_2PO_4.H_2O$), half phosphate (½ P_i; 1.27 g/L K_2HPO_4, 1.30 g/L $NaH_2PO_4.H_2O$; Hypho^{MOD} medium), or quarter phosphate (¼ P_i; 0.63 g/L K_2HPO_4, 0.65 g/L $NaH_2PO_4.H_2O$) and inoculated with 10 µL of cell suspension. Cultures were grown in an Epoch II microplate reader (BioTek, Winooski, VT) for 48 hours, shaking at 548 rpm, incubated at 30°C. Growth of cultures was monitored by measuring light scatter at 600 nm every 30 minutes.

DNA manipulation, Molecular Cloning, and Mutagenesis. The Δ*ppx* mutant strain was generated using the counter selection marker sacB. The donor plasmid was constructed as follows: ~800 bp regions of genomic DNA flanking the *ppx* gene (*META1p2050*) were amplified using primers designed with 20 bp overlaps for homology-based assembly as previously reported [46]. Linearized pCM433KanT was also produced by PCR with 20 bp overlaps for the 5' and 3' flanking regions. The final construct was assembled as previously described [40], Sanger sequenced for verification and transformed into M. extorquens AM1. Counterselection was conducted with 5% sucrose as reported [47]. The deletion was confirmed via PCR and Sanger sequencing.

To prepare plasmid pAZ1 for overproduction of the methylolanthanin biosynthetic gene cluster (*mll*), a yeast in vivo DNA assembly strategy was used based on the DNA assembler method.[1] First, the genes *META1p4132* through *META1p4133* and *META1p4134* through *META1p4138* were PCR-amplified as two 4.5 kb fragments with 400 bp of overlap on each end

from *M. extorquens* AM1 gDNA isolated using the DNeasy PowerMax Soil Kit (Qiagen, Germantown, MD, USA). Two expression elements were obtained using pRES (unpublished) as template: a 4.0 kb E. coli helper fragment (derived from pCC1Fos (Genbank accession EU140751, Epicentre Biotechnologies, Madison, WI, USA) and a 3.3 kb *S. cerevisiae* helper fragment (derived from pYJKSD,(60)). A third fragment (5.0 kb) to integrate elements of expression for *M. extorquens* AM1 was obtained by using pCM66T as a template. To prevent competition with the *E. coli* backbone, the *colE* locus of pCM66T was removed with OE-PCR through amplification of the components up and downstream of *colE* locus. Four different 800 bp joint fragments with 400 bp overlaps with each main fragment were obtained by amplifying expression elements from the *E. coli* fragment, the *S. cerevisiae* fragment, the *mll* gene fragments, and the *M. extorquens* AM1 fragment. Following electrophoresis, PCR products were purified from a 1% agarose gel using the GeneJET Gel Extraction Kit (Thermo Fisher Scientific, Waltham, MA, USA). 150 ng of each PCR product was combined, dried under N2, and the final mixture was resuspended in 4 µL of Milli-Q double-deionized water. Electrocompetent, uracil auxotrophic *S. cerevisiae* HZ848 were freshly prepared and transformed with this mixture and spread on synthetic complete medium minus uracil (SC-ura) plates to select for homologous recombination of the DNA mixture. Eight prototrophic colonies were grown in liquid SC-ura and lysed using the Zymoprep Yeast Plasmid MiniPrep II Kit (Zymo Research, Irvine, CA, USA). The plasmid was purified from lysate using the Zymo Research BAC DNA Miniprep Kit (Zymo Research, Irvine, CA,USA) according to the manufacturer's protocol. The plasmid was then transformed into electrocompetent TransforMax EPI300TM *E. coli* (Lucigen, Middleton, WI, USA), and plated on LB with chloramphenicol for selection. Resultant colonies were grown in liquid SOB media overnight followed by induction through passaging into fresh media with

chloramphenicol and CopyControl Induction Solution (Lucigen, Middleton, WI, USA). After five hours of growth, the plasmid was purified using BAC DNA Miniprep Kit (Zymo Research, Irvine, CA) and verified via Sanger sequencing (UC Berkeley DNA Sequencing Facility, Berkeley, CA, USA). The plasmid was then used in the electroporation of Δ*mxaF* to generate strain Δ*mxaF*/pAZ1.

Processing of smartphones. Postconsumer ZTE Quest N817 and Nokia 6136 smartphones including lithium ion batteries were purchased from eBay. Phones were pulverized using a Blendtec Total Blender (Blendtec, Orum, UT) in pulses of ~20 seconds for 5 to 10 min. The resulting debris was sifted through a set of geological sieves (American Geo, Poughkeepsie, NY). Fragments below 0.15 mm were retained and autoclaved.

Growth analysis with ores, minerals, and smartphones. For growth with ores and minerals, cultures of *M. extorquens* AM1 were grown overnight in MP succinate medium without exogenous lanthanides. When cultures reached mid-log phase (~OD 0.8), a 300 µL aliquot was sub-cultured to 10 mL of fresh MP methanol supplemented with 0.5% ore. As controls, 10 mL MP methanol with ores and without or with the addition of 2 µM lanthanum chloride ($LaCl_3$) was inoculated with 300 µL bacterial aliquots or with MP methanol lacking cells. Every 3 hours, a 200 µL aliquot of culture was removed and OD_{600} was recorded using a Spectramax M2 plate reader (Molecular Devices, Sunnyvale, CA). The no cell control containing the respective ore was used as the blank. For growth with smartphones, 2 mL of MP succinate culture was inoculated into 100 mL MP methanol media with and without 0.5% smartphone powder and $LaCl_3$ in 250 mL shake flasks. Growth was measured by serial dilution and CFU analysis.

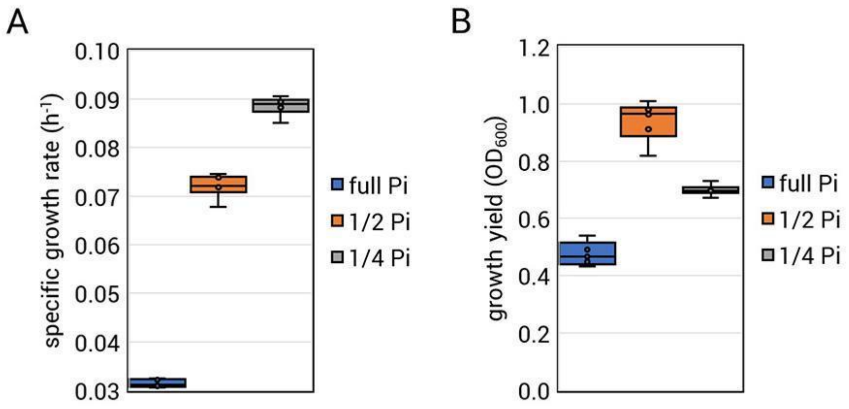

Figure S1. Impact of inorganic phosphate on strain performance. *A,* Growth rate of *M. extorquens* AM1 increases with decreasing inorganic phosphate in the medium. Wild type *M. extorquens* AM1 was grown in a 48-well microplate in Hypho minimal medium with 1.6 g/L methanol and 0.5 mg/L $NdCl_3$. Blue, standard phosphate (P_i; 2.53 g/L K_2HPO_4, 2.59 g/L $NaH_2PO_4.H_2O$). Orange, half phosphate (½ P_i; 1.27 g/L K_2HPO_4, 1.30 g/L $NaH_2PO_4.H_2O$; Hypho^{MOD} medium). Gray, quarter phosphate (¼ P_i; 0.63 g/L K_2HPO_4, 0.65 g/L $NaH_2PO_4.H_2O$). *B*, Yield of *M. extorquens* AM1 grown on methanol with Nd is influenced by inorganic phosphate in the medium. Box plots represent the interquartile ranges for 6 biological replicates; standard deviation is shown by error bars.

Calcium is normally added to Hypho minimal media as an essential metal for MxaF methanol dehydrogenase. However, the strains of *M. extorquens* AM1 used in this study do not have *mxaF* and rely on REE for methanol dehydrogenase activity via XoxF and ExaF. We tested if removing calcium from the growth medium would impact REE uptake in *M. extorquens* AM1 but observed no significant differences (Figure S2).

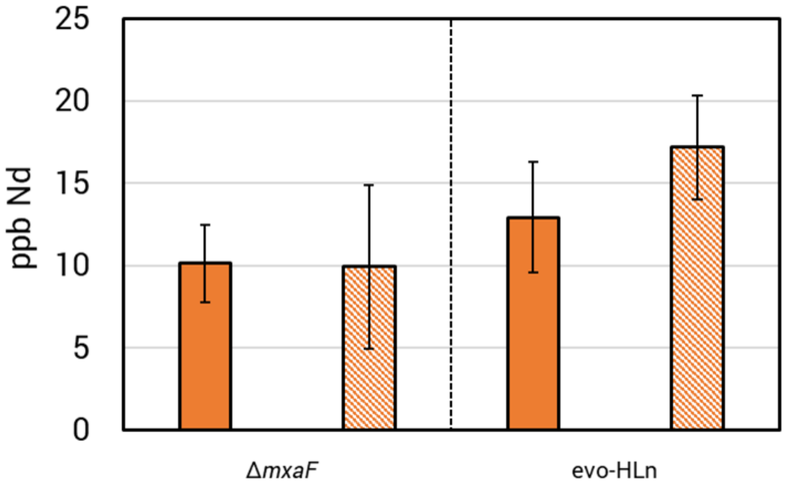

Figure S2. Effect of calcium in growth medium on Nd uptake. *M. extorquens* AM1 Δ*mxaF* and evo-HLn strains were grown in Hypho^{MOD} medium with 1.6 g/L methanol, with (solid bars) or without (hatched bars) 2 μM $CaCl_2$, to early stationary phase. Nd content was determined by ICP-MS and normalized by OD. Bars show the means of three biological replicates; standard deviation is shown by error bars. No significant differences were observed between any pair of conditions at $p < 0.05$ as determined by one-way analysis of variance and Tukey's honestly significant difference test.

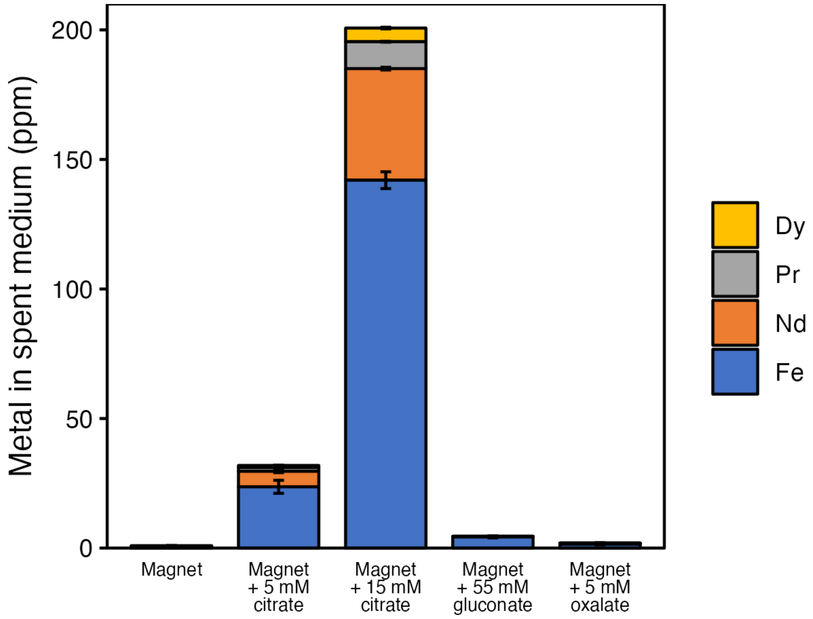

Figure S3. Abiotic leaching of REE from 1% (w/v) magnet swarf in 1 mL Hypho^{MOD} medium supplemented with 5 mM or 15 mM citrate, 55 mM gluconate, or 5 mM oxalate, incubated at 30 °C, as determined by ICP-MS. Each bar and error bars represent mean and standard deviation of triplicate samples, respectively.

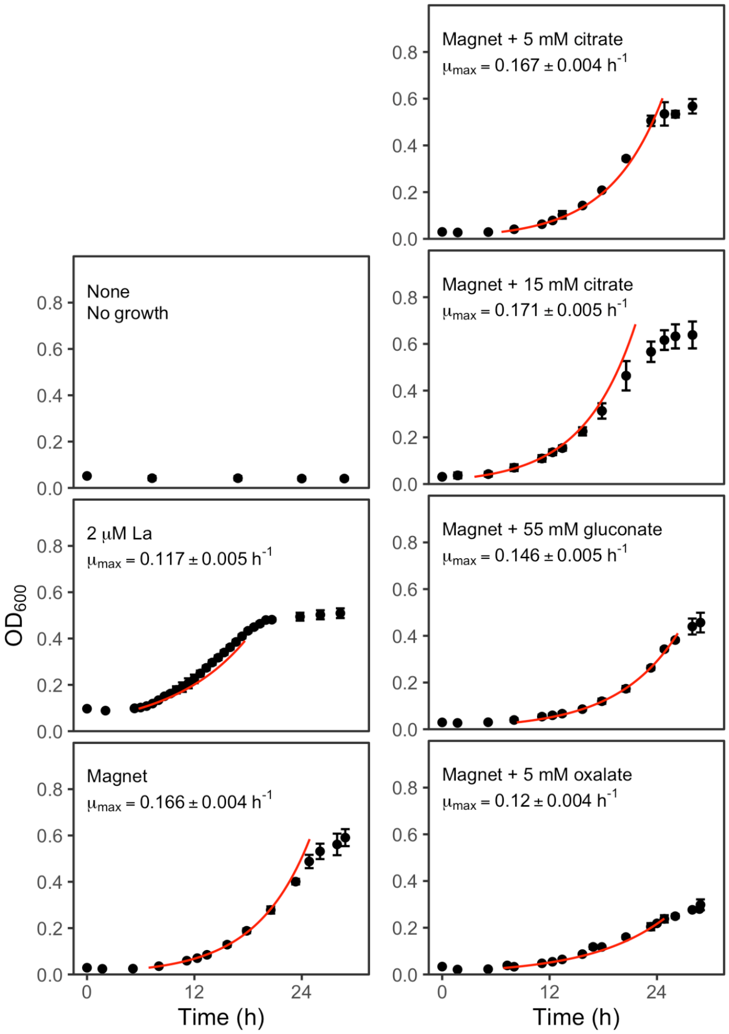

Figure S4. Growth rates of *M. extorquens* AM1 Δ*mxaF* in Hypho^{MOD} medium with 1.6 g/L MeOH, supplemented with La or 1% (w/v) magnet swarf as the source of REE and various organic acids. Each point and error bar represents mean and standard deviation of biological triplicates. The red line represents the exponential growth model fit by calculating the maximum growth rates from the log-linear portion of the growth curves using the function `all_easylinear()` of the R package `growthrates` on at least three biological replicates. Growth experiments were performed in 1-mL volume in 24-well plates.

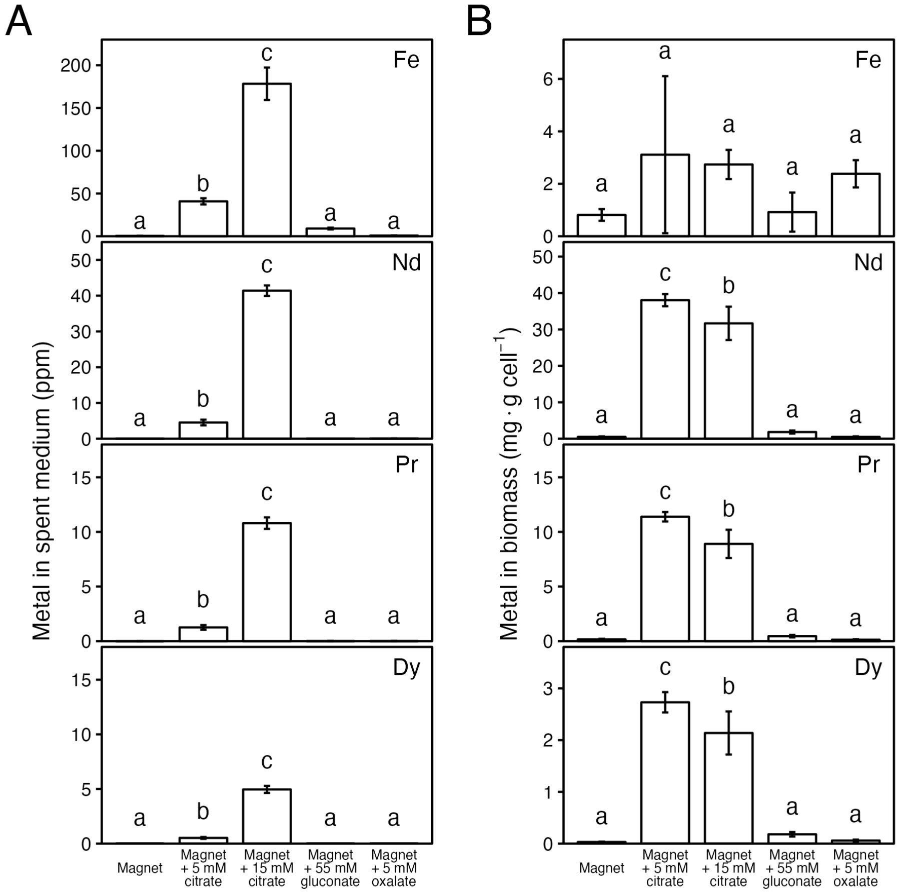

Figure S5. Bioleaching and bioaccumulation during growth with organic acids. Amount of Fe, Nd, Pr, and Dy found in (A) the spent medium and (B) biomass of *M. extorquens* AM1 Δ*mxaF* after growth in 1 mL Hypho^{MOD} with 1.6 g/L MeOH and 1% (w/v) magnet swarf, supplemented with 5 mM or 15 mM citrate, 55 mM gluconate, or 5 mM oxalate. Metal concentrations were determined by ICP-MS. Bars and error bars represent mean and standard deviation of triplicate samples. Letters above bars represent groups of statistical significance as determined by ANOVA followed by Tukey HSD test ($p < 0.05$).

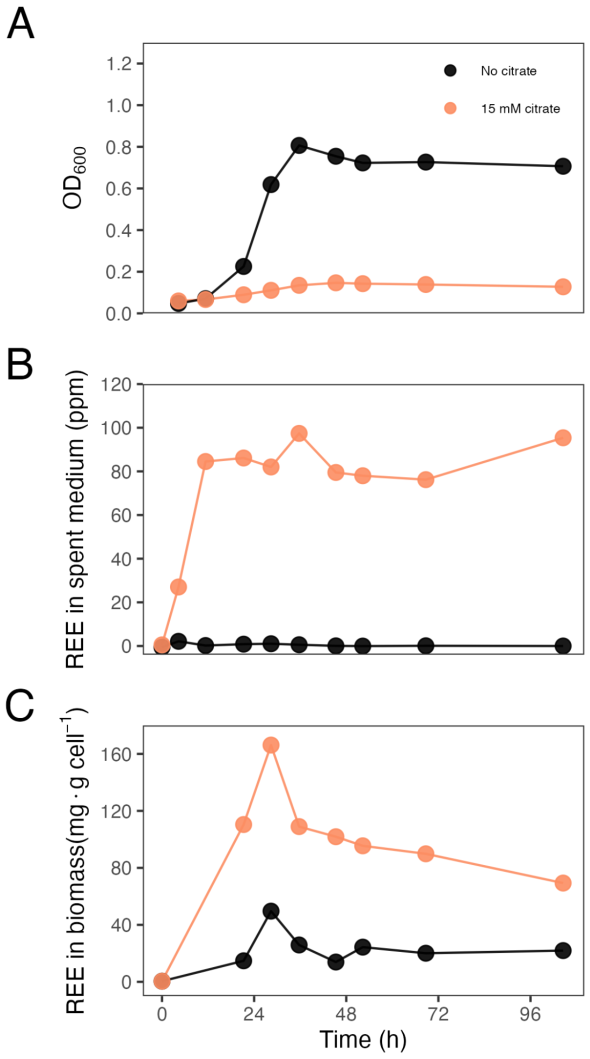

Figure S6. Effect of citrate on bioleaching and bioaccumulation. (A) Growth of *M. extorquens* AM1 Δ*mxaF* in 100 mL of Hypho^{MOD} medium with 1.6 g/L MeOH, with and without 15 mM citrate. Concentrations of REE in (B) spent medium and (C) cell pellets were analyzed using the Arsenazo III assay. Each point represents one biological replicate.

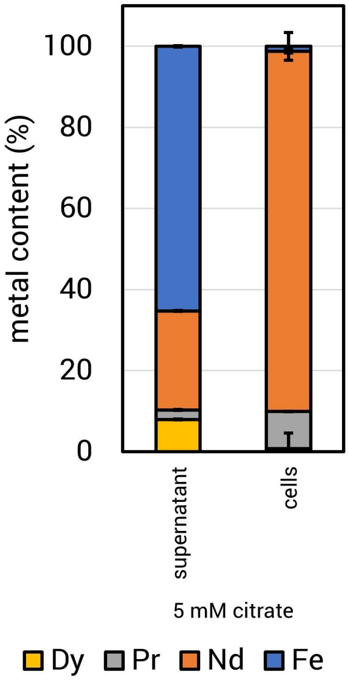

Figure S7. Selective bioconcentration of REE from culture grown in a 0.75 L benchtop bioreactor. *M. extorquens* AM1 was grown in Hypho^{MOD} methanol medium with 1% Nd magnet swarf and 5 mM citrate. Cell and supernatant samples were taken once the culture reached early stationary growth phase. Metal concentrations in samples were determined by ICP-MS and normalized to the total metal uptake. Plots show the means of three independent growth experiments with error bars showing standard deviations. Dy, dysprosium; Pr, praseodymium; Nd, neodymium; Fe, iron.

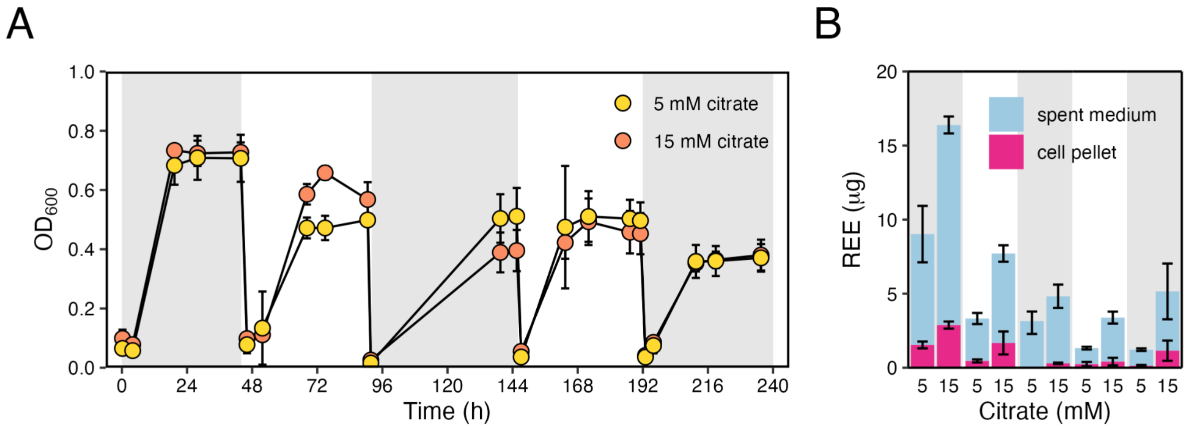

Figure S8. Iterative bioaccumulation from a single swarf batch. Growth of *M. extorquens* AM1 Δ*mxaF* in 1 mL of Hypho^{MOD} medium with 1.6 g/L MeOH, 1% (w/v) magnet swarf, and varying concentrations of citrate. (A) Five cycles of growth were repeated for the same magnet swarf sample by replacing culture with fresh medium, then (B) REE in spent medium and cell pellet analyzed via Arsenazo III assay. Each point or bar represents the mean of biological triplicates, and error bars the standard deviation.

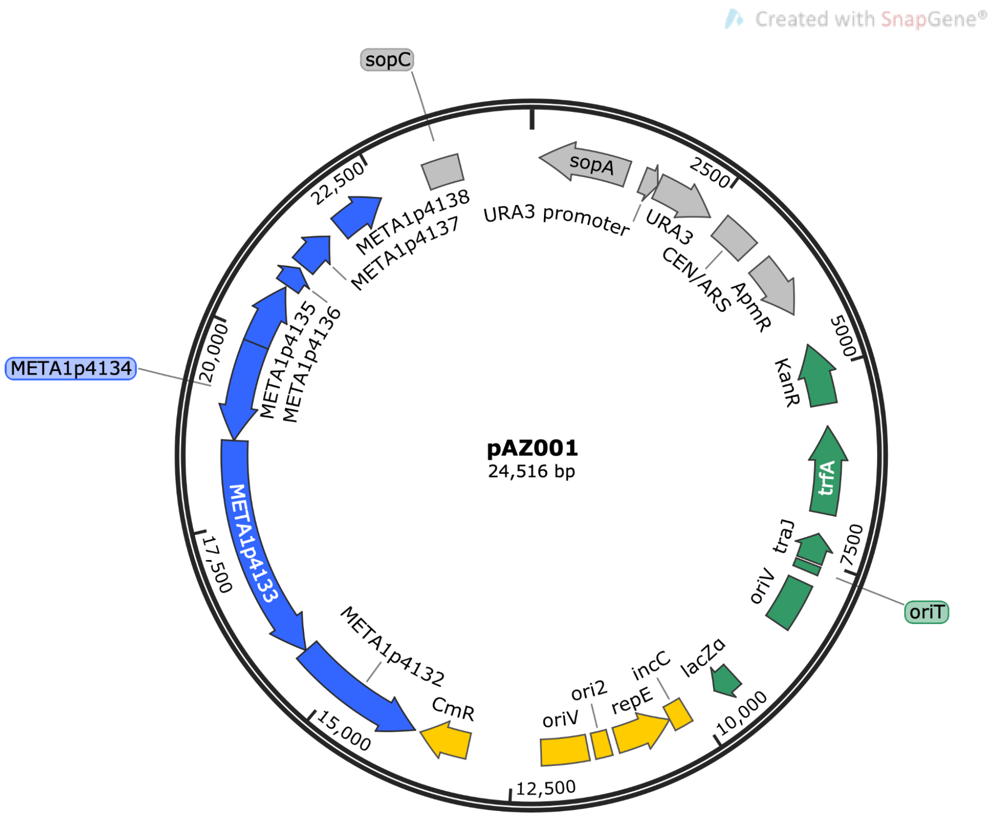

Figure S9. Plasmid map of pAZ1 with *mll* genes (*META1p4129* through *META1p4138,* blue) and segments for expression in *S.cerevisiae* (grey), *M. extorquens* (green), and *E. coli* (yellow).

SUPPORTING TABLES

Table S1. Ratio of metals chemically leached from magnet swarf in the presence of citrate, gluconate, and oxalate as compared to the absence of chelator (mean ± SD).

	Fe	Nd	Pr	Dy
Magnet (reference)	1.0 ± 0.6	1.1 ± 0.3	1.0 ± 0.3	1.2 ± 0.5
Magnet + 5 mM citrate	67.4 ± 7.2	15.8 ± 1.4	13.0 ± 0.9	33.5 ± 5.1
Magnet + 15 mM citrate	404.4 ± 9.2	113.8 ± 1.4	92.3 ± 1.5	223.0 ± 13.6
Magnet + 55 mM gluconate	12.1 ± 0.9	0.6 ± 0.2	0.6 ± 0.2	0.7 ± 0.3
Magnet + 5 mM oxalate	4.2 ± 0.5	1.0 ± 0.4	1.0 ± 0.4	1.6 ± 0.6

Table S2. Growth of *M. extorquens* AM1 with pure and complex REE sources

REE source	growth rate
2 μM $LaCl_3$	0.16 ± 0.01
0.5% monazite ore	0.15 ± 0.03
0.5% bastnasite ore	0.16 ± 0.00
0.5% bastnasite crystal	0.16 ± 0.01
0.5% blended smartphone	0.16 ± 0.01[ϵ]

[ϵ]growth rate determined from cfu measurements every 4 h.

Flexibility of M. extorquens for REE bioleaching and bioaccumulation from complex sources

Robust growth and bioaccumulation of Nd from magnet swarf indicated that *M. extorquens* AM1 can effectively leach and acquire Ln from poorly soluble sources. Therefore, we tested the ability of *M. extorquens* AM1 to grow with unrefined REE sources with very low solubility in the growth medium. Monazite ore, for example, contains up to ~45% REEs, with the Ce_2O_3 content comprising as much as 17% of the total REEs. However, the REEs occur as poorly soluble phosphates and fluorocarbonates in these ores. When challenged with the ores monazite or bastnäsite as the sole REE source, *M. extorquens* AM1 grew as well as with soluble $LaCl_3$ (Table 2). Similar growth results were also observed with bastnäsite crystal (Table S2).

Complex debris like E-waste represents virtually untapped reservoirs of valuable REEs. Cellular smartphones contain several species of REE, including yttrium, lanthanum, terbium, neodymium, gadolinium and praseodymium, making smartphone E-waste a valuable, unutilized source for REE recovery. However, smartphone E-waste poses two major challenges for

bioleaching. The metals in this E-waste are highly insoluble in oxide form, and smartphone batteries contain other metals (*e.g.,* iron, boron, copper, tellurium, manganese, mercury, lead, tungsten, lithium, cobalt), all of which can be toxic. We tested the feasibility of using smartphone E-waste as a REE source by assessing the capacity of *M. extorquens* AM1 to grow on methanol. Somewhat surprisingly, with 0.5% pulp density (w/v) of blended smartphones, *M. extorquens* AM1 grew as well as with soluble $LaCl_3$ (Table S2). In fact, along with the ores and crystal REE sources, the insolubility of the metals and complexity of the source did not significantly impact growth rate or growth yield of the culture (Table S2) showing the flexibility and robustness of *M. extorquens* AM1 as a biological platform for the bioaccumulation of REE from complex sources.

REFERENCES

(1) Shao, Z.; Zhao, H.; Zhao, H. DNA Assembler, an in Vivo Genetic Method for Rapid Construction of Biochemical Pathways. *Nucleic Acids Res.* **2009**, *37* (2), e16. https://doi.org/10.1093/nar/gkn991.
